# Towards a 'chassis' for bacterial magnetosome biosynthesis: genome streamlining of *Magnetospirillum gryphiswaldense* by multiple deletions

**DOI:** 10.1186/s12934-021-01517-2

**Published:** 2021-02-04

**Authors:** Theresa Zwiener, Marina Dziuba, Frank Mickoleit, Christian Rückert, Tobias Busche, Jörn Kalinowski, René Uebe, Dirk Schüler

**Affiliations:** 1 Department of Microbiology, University of Bayreuth, Bayreuth, Germany; 2 Institute of Bioengineering, Research Center of Biotechnology of the Russian Academy of Sciences, Moscow, Russia; 3 Center for Biotechnology, University of Bielefeld, Bielefeld, Germany

**Keywords:** *Magnetospirillum gryphiswaldense*, Magnetotactic bacteria, Magnetosomes, Genome reduction, Chassis, IS elements

## Abstract

**Background:**

Because of its tractability and straightforward cultivation, the magnetic bacterium *Magnetospirillum gryphiswaldense* has emerged as a model for the analysis of magnetosome biosynthesis and bioproduction. However, its future use as platform for synthetic biology and biotechnology will require methods for large-scale genome editing and streamlining.

**Results:**

We established an approach for combinatory genome reduction and generated a library of strains in which up to 16 regions including large gene clusters, mobile genetic elements and phage-related genes were sequentially removed, equivalent to ~ 227.6 kb and nearly 5.5% of the genome. Finally, the fragmented genomic magnetosome island was replaced by a compact cassette comprising all key magnetosome biosynthetic gene clusters. The prospective 'chassis' revealed wild type-like cell growth and magnetosome biosynthesis under optimal conditions, as well as slightly improved resilience and increased genetic stability.

**Conclusion:**

We provide first proof-of-principle for the feasibility of multiple genome reduction and large-scale engineering of magnetotactic bacteria. The library of deletions will be valuable for turning *M. gryphiswaldense* into a microbial cell factory for synthetic biology and production of magnetic nanoparticles.

## Background

Magnetosomes are membrane-enclosed organelles that are synthesized by various aquatic bacteria for their magnetotactic navigation in the Earth’s geomagnetic field [1, 2]. Apart from their biological function as magnetic sensors, magnetosomes also represent microbially synthesized magnetic nanoparticles (MNP) consisting of monocrystalline magnetite (Fe_3_O_4_) or greigite (Fe_3_S_4_). Because of their strictly controlled biomineralization, bacterial magnetosomes have exceptional properties, such as high chemical purity and crystallinity, strong magnetization, and uniform sizes and shapes, which are largely unknown from chemically synthesized MNP [3–5]. This makes them highly attractive for a number of biotechnological and biomedical applications [6–8]. For examples, magnetosomes isolated from the magnetic bacterium *Magnetospirillum gryphiswaldense* were successfully tested as multimodal reporters for magnetic imaging [9, 10], nanocarriers for magnetic drug targeting [11–13], and for magnetic hyperthermia applications [14, 15]. In addition, the functionality of magnetosomes can be extended by genetically fusing foreign functional moieties and polypeptides, such as fluorophores, enzymes, antibodies, and organic shells [16–22] to magnetosome membrane anchors. Moreover, the bacteria were utilized as a model to study the molecular mechanisms of human diseases related to homologs of certain magnetosome proteins [23].

However, applications of bacteria and their magnetosomes so far have been hampered by the limited number of appropriate production strains and difficulties in their large-scale cultivation and genetic manipulation. One of the most extensively investigated model organisms for studying magnetosome biosynthesis is the freshwater alphaproteobacterium *M. gryphiswaldense* [24, 25]. It typically produces 15–25 cuboctahedral magnetite particles per cell that are about 40 nm in size [26], while genetic overexpression of gene clusters governing magnetosome biosynthesis generated an overproducing strain forming > 100 (up to 170) particles per cell with enlarged sizes [27]. Because of its genetic tractability and relatively straightforward cultivation, *M. gryphiswaldense* recently has also emerged as host strain for bioproduction and synthetic biology of magnetosomes [20, 21, 27–31].

Despite of this recent progress, since its isolation [24, 32, 33] the usability of the undomesticated *M. gryphiswaldense* as a biotechnological workhorse has been limited due to several unwanted features. For example, one obstacle is the rather fastidious and sometimes fluctuating growth, which makes cultivation difficult to reproduce at larger scale. In other bacteria, this erratic growth behavior has been attributed to the presence of prophage genes, which are often known to exhibit some latent activity, resulting in a negative impact on the robustness of growth and the performance of bioprocesses [34–36]. Another adverse feature is the inherent genetic instability of *M. gryphiswaldense*, and in particular of the magnetic phenotype, which makes genetic manipulation and magnetosome production cumbersome. For example, spontaneous loss or impairment of magnetosome biosynthesis has been observed frequently during subcultivation, which had been traced back to spontaneous deletions and rearrangements within the large genomic **ma**gnetosome **i**sland (MAI) [37–39]. This chromosomal region extends across about 100 kb and comprises discontiguous clusters of more than 30 genes responsible for magnetosome formation organized in the five polycistronic operons *feoAB1*, *mms6*, *mamGFDC*, *mamAB* and *mamXY* [1, 40]. In addition, the MAI harbors regions of irrelevant gene content and numerous mobile genetic elements, which might be responsible for the frequent rearrangements and loss of magnetic phenotype in *M. gryphiswaldense* [37, 38, 41].

For future synthetic biology applications as well as large-scale magnetosome bioproduction, a simplified and potentially more robust version of the *M. gryphiswaldense* genome would be highly beneficial. In other bacteria, moderate genome reduction, which comprises the targeted deletion of multiple dispensable genes, has been shown to optimize metabolic pathways, enhance the expression of recombinant protein productivity, and improve physiological performance and growth [42–46]. For instance, by removing non-essential, recombinogenic or mobile DNA and cryptic virulence genes, genome reduction of *Escherichia coli* resulted in several favorable properties, such as increased electroporation efficiency and improved propagation of recombinant genes [47]. Deletion of prophage genes improved growth and transformation efficiency in *Corynebacterium glutamicum* [34], enhanced genotypic stability in *Pseudomonas putida* [35, 48, 49], and increased robustness toward stress in *Vibrio natriegens* or *Shewanella oneidensis* MR-1 [36, 50]. Furthermore, deletion of active mobile genetic elements caused enhanced protein productivity in *C. glutamicum* [51], and increased transformability and reduced mutation rates in *Acinetobacter baylyi* [52].

In magnetotactic bacteria, comparable genome reduction approaches so far have been impeded because of the unavailability of efficient methods for large-scale engineering of these recalcitrant microorganisms. To overcome these current limitations, we recently started a systematic approach to engineer the model strain *M. gryphiswaldense* at the genome level. In a previous study, we established a method for large-scale deletion mutagenesis and utilized it for the generation of 24 single deletions covering about 167 kb of non-redundant genome content. We thereby identified large regions inside and outside the MAI irrelevant for magnetosome biosynthesis [53]. Here, we continued our work by constructing genome-reduced strains of *M. gryphiswaldense* with multiple combinatorial deletions of irrelevant and detrimental gene content. We provide a proof of concept for large-scale genome editing and improvement towards a future chassis [54], which may turn *M. gryphiswaldense* into a microbial cell factory for the synthetic biology and high-yield production of magnetic nanoparticles.

## Results

### Overview over the experimental strategy

**Fig. 1:**
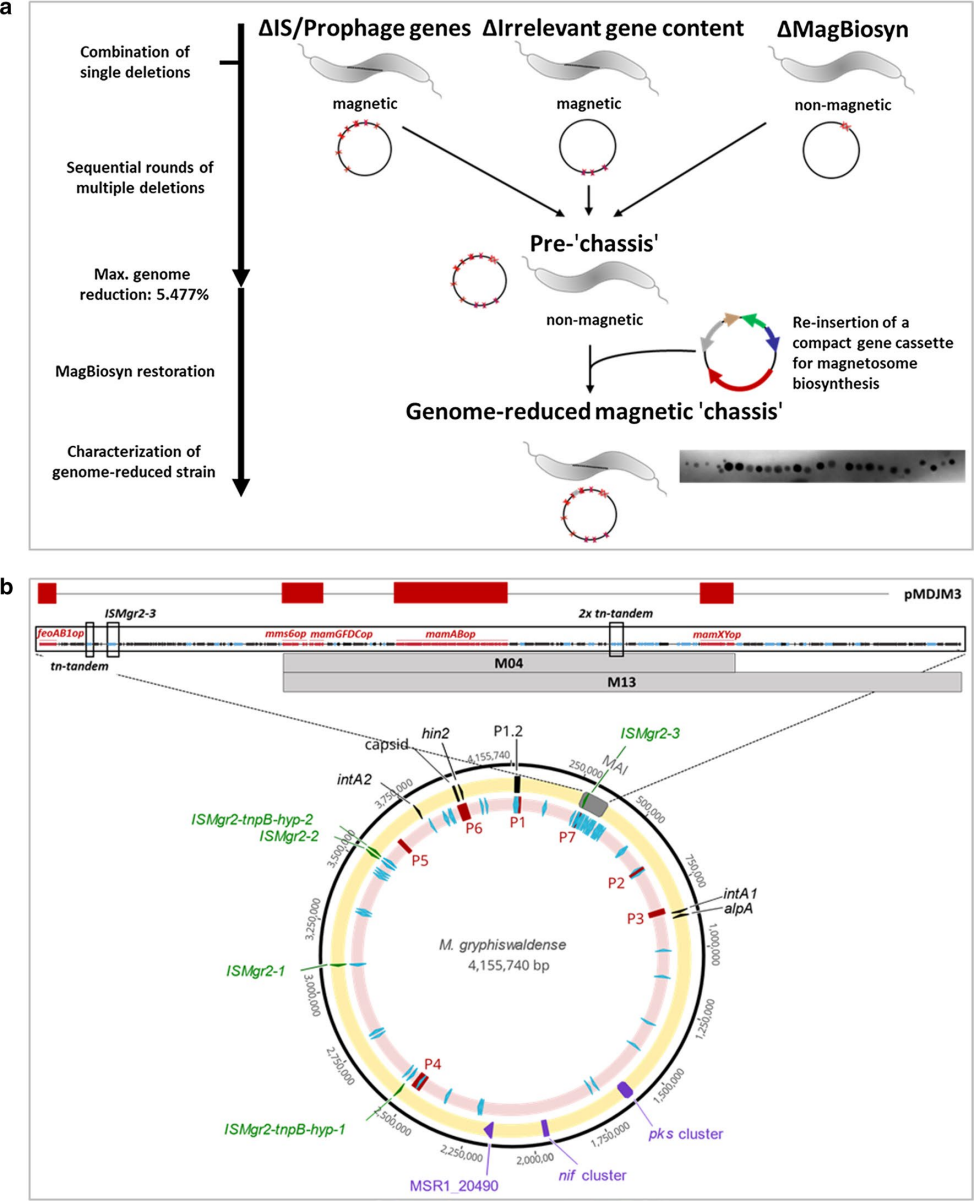
Overview over the experimental workflow (**a**), and the genomic positions of deletion targets in *M. gryphiswaldense* (**b**). Yellow circle (**b**) shows genes or gene sets targeted for multiple deletions. Grey: magnetosome island (MAI); black arrows: parts of predicted prophage sets and phage-related integrase and excisionase genes; green arrows: insertion element genes identified as most active in this study; purple: irrelevant gene clusters. Pink circle (**b**) indicates predicted prophage sets (dark red) and mobile genetic elements (light blue). The enlarged area shows the genetic organization of the native MAI with all five known magnetosome biosynthesis operons (red), genes of known or unknown function irrelevant for magnetosome formation (black), mobile genetic elements (light blue). The grey bars indicate the extent of regions M04 (~ 65 kb) and M13 (~ 100 kb), which were shown to be deletable en bloc in our previous study [53]. The presence of key magnetosome biosynthesis genes within the compact expression cassette of the applied vector pMDJM3 is indicated by red bars while connecting lines designate eliminated gene content

The features of a genome reduced future 'chassis' should include first the elimination of problematic and harmful gene content such as prophage genes as well as active IS elements known to cause genetic instability [50–52]. Second, the genome should be freed of as much of gene content unnecessary for magnetosome biosynthesis, growth and fitness under lab conditions as possible. Third, neutral and favorable scarless deletions should be combined into one or few single strains. Ultimately, the native biosynthetic gene clusters within the MAI plus multiple large portions of 'junk' between and adjacent to them should be substituted by a compact cassette comprising all key genes for magnetosome biosynthesis (plasmid pMDJM3). Final strains were tested for growth, fitness, and genetic stability (for an overview over the experimental workflow see Fig. 1a).

### Identification and elimination of prophage genes

**Fig. 2:**
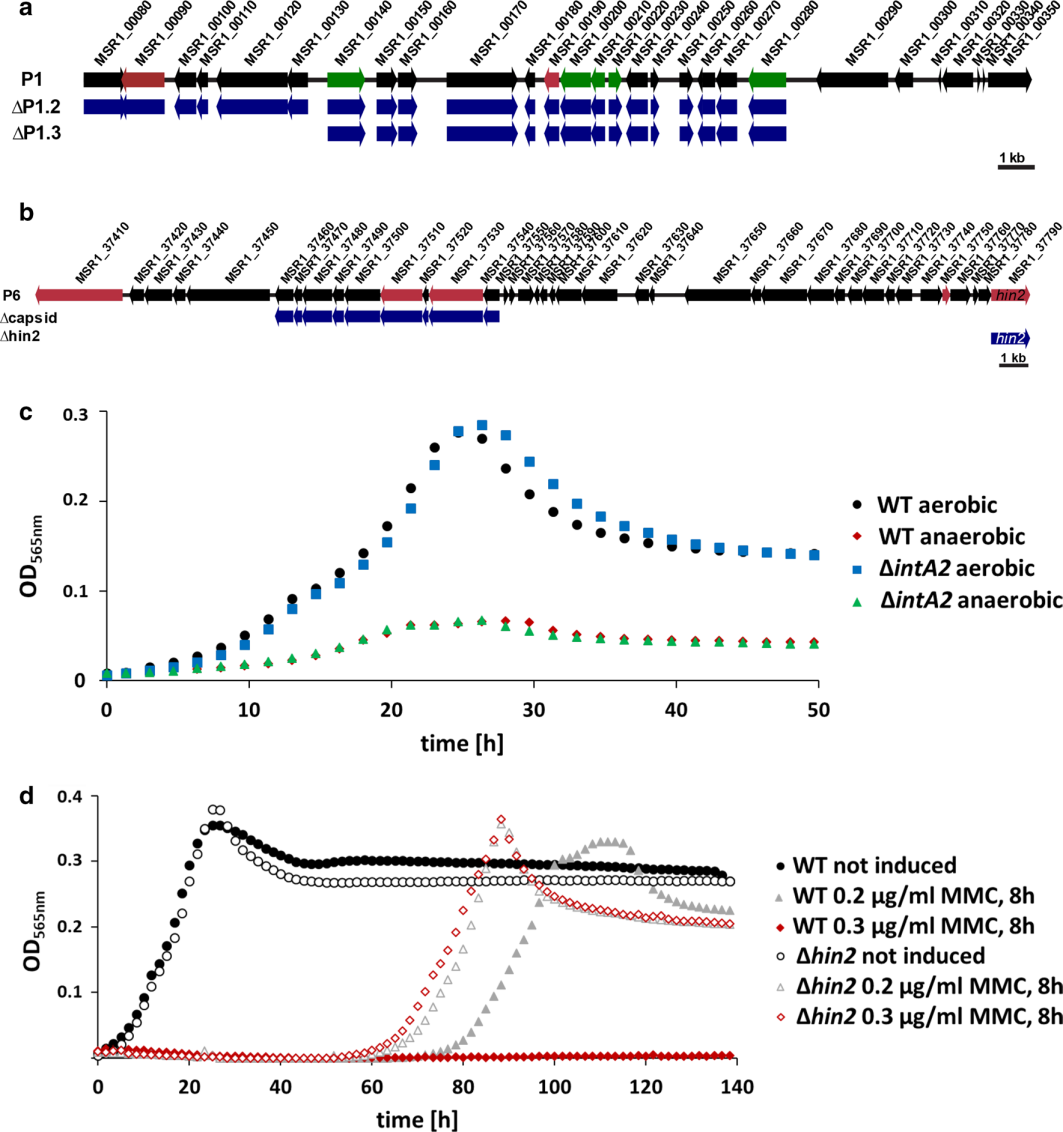
Molecular organization and analysis of predicted prophages P1 (**a**), P6 (**b**), deleted genes and growth characteristics of selected cultures (**c**, **d**). **a**, **b** Black: hypothetical genes or genes with known function; red: putative prophage genes; green: mobile genetic elements; blue: deleted genes. **c** Growth characteristics of ∆*intA2* under aerobic and anaerobic conditions compared to WT. Growth of other prophage deletants (not shown) was virtually identical. **d** Growth profiles of mitomycin C-treated (MMC) cultures of a selected single prophage deletion mutant and the WT. Cultures were incubated for 8 h at MMC concentrations of 0.2 or 0.3 µg/ml, washed twice, and adjusted to the initial OD. Growth experiments were performed at 28 °C under aerobic conditions. Each strain was analyzed in triplicates, the curve represents the calculated average (standard deviations were < 5%)

Since its isolation [24, 32, 33], our lab persistently experienced occasional problems with the cultivation of *M. gryphiswaldense*, such as poorly reproducible and fluctuating growth, which could not only be explained by unintended subtle variations in handling, media constituents and incubation alone. In other bacteria, similar observations could be traced back to the latent activity and induction of prophages, which are known to often have a negative impact on robustness of growth and the performance of bioprocesses [34]. In the genome of *M. gryphiswaldense*, we detected seven putative prophage regions (referred to as P1–P7) (Fig. 1b) by the phage search tool PHAST [55]. Two of them (P1, 26 kb, and P6, 34.1 kb) were predicted as intact (Figs. 1b and 2a, b), but upon closer inspection only P6 is contiguous and seems to have a full complement of typical phage genes (e.g. integrases, tail, and major capsid proteins), whereas P1 is interspersed with tRNA genes (Fig. 2a, b). Predicted prophage P2 seems to be incomplete as well, consistent with its small size (7.2 kb) and its accumulation of several transposon genes. Region P3 (20 kb) comprises some putative essential genes (e.g. encoding transcriptional regulators, chaperones) and a phage integrase *intA1.* Incomplete P4 (31.2 kb) and P5 (14.3 kb) are also interspersed with genes of unrelated, but important functions, e.g. chaperones of DnaJ-class, a transcriptional regulator and a ribonuclease, respectively. Incomplete P7 (11.3 kb) resides inside the part of the MAI (Fig. 1b) that was non-deletable in previous experiments [53]. From the identified putative phages, the following regions were selected as targets for deletion: P1 was divided into two parts excluding the essential tRNA genes (P1.2, 19.4 kb, and P1.3, 12.8 kb) that were both deleted separately (Fig. 2a). Since deletion of whole P6 failed, only genes encoding putative capsid proteins (7.65 kb) and a recombinase (*hin2*, 1.34 kb) were deleted separately (Fig. 2b). Furthermore, inside and adjacent to these predicted prophages, we identified several putative integrases and excisionases that might be involved in the reactivation of lysogenic prophages to the lytic cycle and decided to delete several candidates as well (*intA1* 1.28 kb, *intA2* 1.22 kb and *alpA* 225 bp) (Fig. 1b).

Under aerobic and anaerobic conditions, growth of all prophage deletants was largely indistinguishable from the WT (data not shown). After incubation with 0.2 µg/ml MMC, which is known to trigger the cellular SOS response and to induce prophages to enter the lytic cycle [36, 56], growth was indistinguishable from the WT for most deletants. A notable exception was ∆*hin2* (*msr1_37790*), which proved to be less sensitive and could be re-grown after incubation with up to 0.3 µg/ml MMC (Fig. 2c).

### Deletion of active mobile genetic elements

**Fig. 3:**
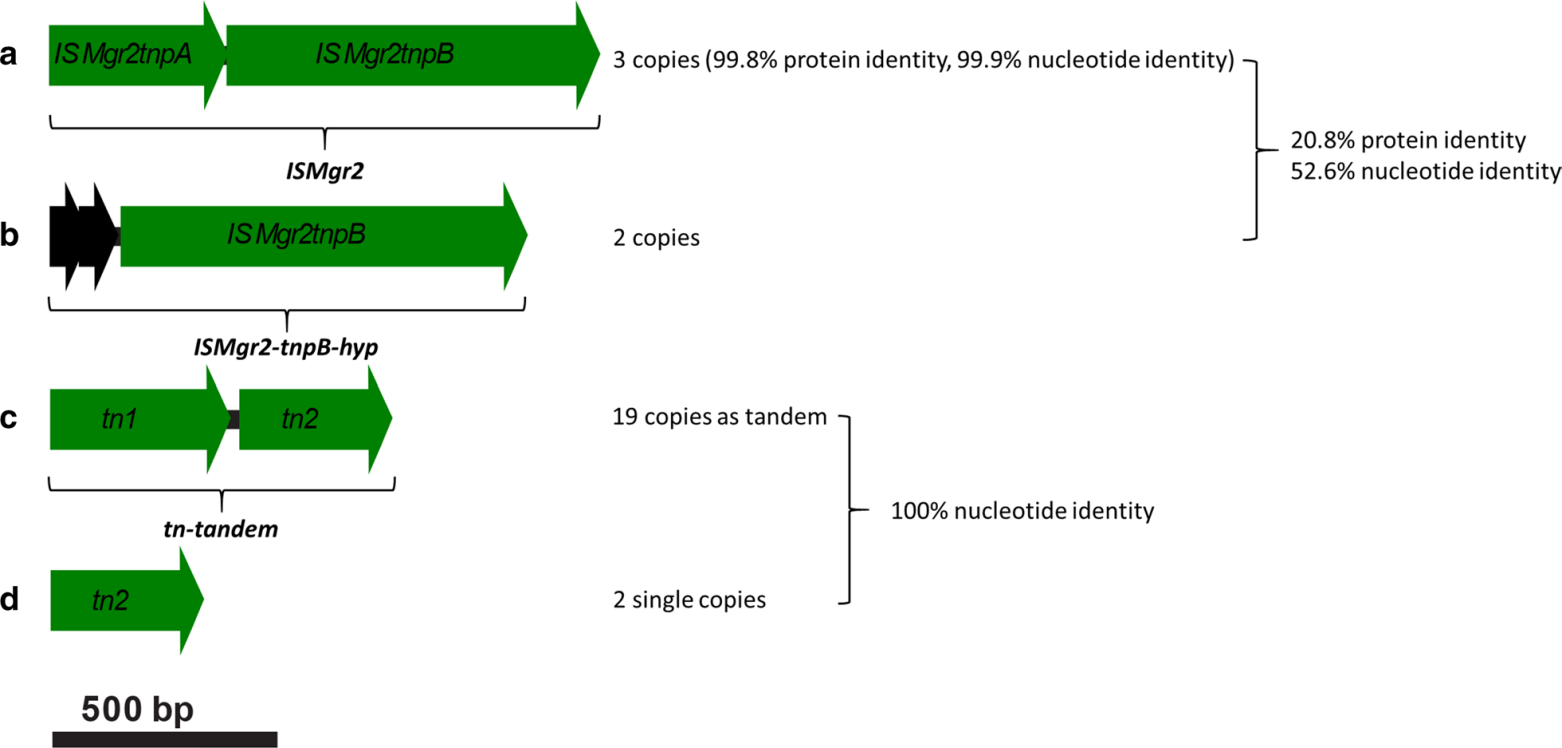
Overview over identified active mobile genetic elements in *M. gryphiswaldense*. **a** One group of active insertion elements (*ISMgr2tnpA* and *ISMgr2tnpB*, green) belongs to the IS3 family, all three copies were deleted in this study. **b** The second variant of this group contains two hypothetical genes (black) and an *ISMgr2tnpB* gene. Both copies were deleted. **c** A further group of active mobile elements is represented by a transposon tandem (*tn-tandem*). The first transposon (*tn1*) of the *tn-tandem* is a member of the putative transposase of IS4/5 family and contains a DUF4096 domain known to bind the end of a transposon and to catalyze the movement of the transposon to another part of the genome by cut and paste or replicative transposition mechanism. The second *tn-tandem* transposon (*tn2*) contains a DDE domain, named after a conserved amino acid triad Asp, Asp, Glu, the active site [57]. **d** *tn2* is present in two single copies in the WT genome. Two copies of *tn-tandem* were deleted in ∆TZ-16::MAG-*gusA* and ∆TZ-17::MAG-*gusA*

**Fig. 4:**
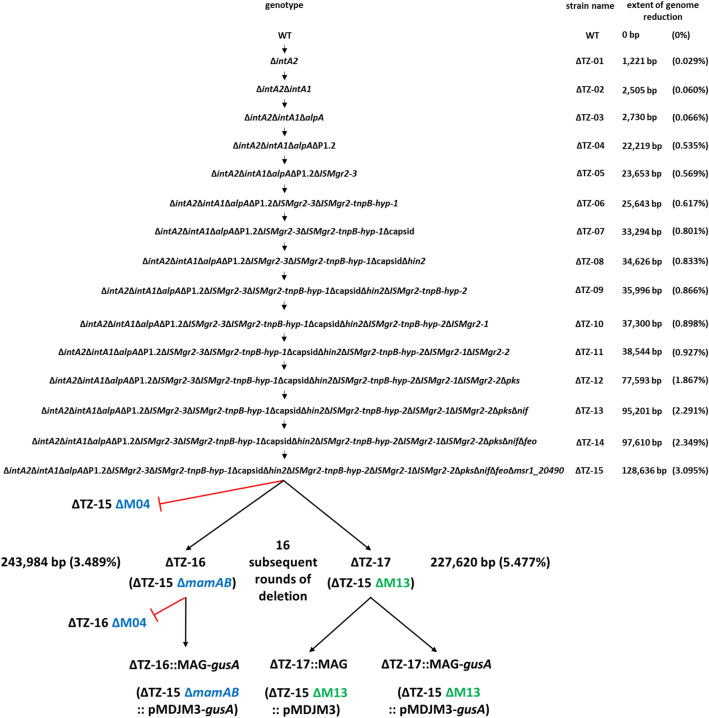
Scheme of multiple deletions and their genotypes. Black arrows indicate successful deletions while red bars show failed deletions. Colored letters indicate final strains with ∆*mamAB* (blue) or ∆M13 (green) deletions. Extents of genome reduction of multiple deletion mutants are given in bp and % of the WT genome

Previous observations had revealed a genetic instability of the *M. gryphiswaldense* WT strain: first, the ability to form magnetosomes often became spontaneously reduced or lost entirely, which had been hypothesized to be caused by the presence and activity of numerous mobile elements, resulting in insertions by transposition activity [53], plus deletions and rearrangements caused by homologous recombination between identical copies [37, 38, 41]. Second, during the course of routine genetic manipulation, we frequently also observed spontaneous inactivation of introduced foreign genes, such as chromogenic reporters (e.g. *gusA*, unpublished observations) or genetic markers for antibiotic or counterselection (e.g. *galK*) [53]. Our preliminary analysis revealed that inactivation was often due to insertion of mobile genetic elements belonging to two types, each with two variants: the first type is a bipartite insertion element (in the following referred to as *ISMgr2*; Fig. 3a), composed of genes encoding a putative IS2 repressor TnpA, and an IS2 transposase TnpB, respectively. *ISMgr2* belongs to the IS3 family that is common in many α-Proteobacteria [57]. Three copies of *ISMgr2* (*ISMgr2-1*, *ISMgr2-2* and *ISMgr2-3*) with 99.8% protein identity (99.9% nucleotide identity) are present in the genome of *M. gryphiswaldense* (Fig. 3a), with one of them residing within the MAI (Fig. 1b, *ISMgr2-3*). Two additional homologs of *tnpB*, termed *ISMgr2-tnpB-hyp-1* and *ISMgr2-tnpB-hyp-2,* with lower (20.8%) protein identity (52.6% nucleotide identity) compared to the first three copies *ISMgr2-1*, *ISMgr2-2* and *ISMgr2-3* (Fig. 3b) could be identified. Each of these latter two homologs are associated with two conserved hypothetical genes upstream of the IS2 transposon gene *tnpB* instead of *tnpA*. We first deleted each of the five homologs individually in the WT background (1–1.25 kb each). As expected, single deletion mutants of all five strains displayed WT-like growth and magnetosome biosynthesis under aerobic and anaerobic conditions, and under oxidative and moderate heat stress (data not shown). We therefore later decided to sequentially delete these five homologs altogether (see below strains ∆TZ-05, ∆TZ-06, ∆TZ-09, ∆TZ-10 and ∆TZ-11 in Fig. 4).

The second type of active mobile elements is represented by a transposon tandem (*tn-tandem*) of genes sharing 52.3% nucleotide identity (31.25% protein identity) (Fig. 3c): its first gene (*tn1*) encodes a putative transposase of the IS4/5 family [57], and the second gene (*tn2*) a DDE domain transposase [57] (Fig. 3c). This tandem pair is present in the genome of *M. gryphiswaldense* in 19 identical (100% nt) copies, and in addition *tn2* alone in two more identical single copies. Four of the 19 *tn-tandem* pairs reside within the MAI, with two of them comprised within the deletable regions M04 and M13 [53] (Fig. 1b). However, due to the unavailability of multiplex genetic tools for *M. gryphiswaldense*, the high copy number of the *tn-tandem* pairs proved prohibitive for sequential or simultaneous deletion of all copies.

#### Identification and elimination of further gene clusters irrelevant for cell growth and magnetosome biosynthesis

Next, to eliminate further larger non-essential chromosomal stretches outside the MAI, we exemplary targeted two gene clusters that are likely irrelevant for magnetosome biosynthesis and fitness in lab conditions (Additional file 1: Table S1): (i) A *nif* operon comprising 16 genes, namely *nifWABZTHDK*, *fixABC*, *draGT* and three ferredoxin genes (*msr1_18560*; *msr1_18600*; *msr1_18640*). The *nif* operon is likely linked to nitrogen fixation in *M. gryphiswaldense* [58] (Additional file 1: Fig. S1) which is irrelevant under the denitrifying conditions optimal for magnetosome biosynthesis [28]. We generated a mutant in which ~ 20 kb of this *nif* cluster comprising 20 genes were deleted (Additional file 1: Fig. S1). (ii) Several uncharacterized clusters encoding a putative non-ribosomal peptide synthetase (NRPS) and a polyketide synthase (PKS) were predicted [59]. Since it was unlikely to be necessary for magnetosome biosynthesis, we deleted three large ORFs encoding putative PKS proteins from one of the clusters (termed *pks*) extending over ~ 40 kb (Fig. 1b; Additional file 1: Table S1). As expected, strains harboring single deletions in *nif* or *pks* clusters were indistinguishable from the WT with regard to magnetosome formation, cell growth and motility, which confirmed their irrelevance for magnetosome biosynthesis and fitness under lab conditions (Additional file 1: Table S1, Fig. S4).

### Combinatory mutagenesis

In order to combine all previously tested favorable or neutral deletions into one or two single strains, we employed the following strategy (Fig. 4): starting with the ∆*intA2* strain as a parent, we first proceeded by deleting further selected prophage genes (∆TZ-01–∆TZ-04), then continued with the mobile genetic elements (∆TZ-05 and ∆TZ-06) and further prophage genes and IS elements (∆TZ-07–∆TZ-11) and ended with deletion of irrelevant gene clusters and magnetosome biosynthesis genes (∆TZ-12–∆TZ-17). One round of deletion was completed as soon as the loss of kanamycin resistance marker (Km^r^) was verified by replica plating. After each round, magnetic responses of mutant strains as well as their growth under aerobic and anaerobic conditions, oxidative and moderate heat stress were tested. This was found to be WT-like for all offspring strains including ∆TZ-15 (Additional file 1: Fig. S2).

After fifteen successful rounds of deletions, the resulting mutant ∆TZ-15 was used as a parent to delete large parts of the MAI including all magnetosome biosynthesis operons. In a previous study, a contiguous stretch of ~ 66 kb termed region M04 was found to be deletable in the WT-background, including all *mam* and *mms6* operons (~ 27 kb) plus ~ 39 kb of irrelevant or problematic gene content, such as two copies of *tn-tandem* (Fig. 1b, enlargement, grey bar) [53]. This had no obvious effects on growth, and magnetosome biosynthesis could be restored to WT-level by complementation with a compact expression cassette comprising the *mam* and *mms6* operons only [53]. In addition, a ~ 100 kb region termed M13 (Fig. 1b) could be excised, again including all *mam* and *mms6* operons, plus an additional ~ 33 kb flanking region. Despite of its slightly impaired growth in oxidative stress conditions [53], the M13 region was chosen as an additional target to generate a strain with the largest possible genome reduction.

However, we failed to delete M04 in strain ∆TZ-15 despite of several attempts (Fig. 4), although its deletion had been readily achieved before in the WT background [53]. Instead, upon repeated attempts of conjugation and counterselection, we obtained a number of conspicuous clones with either magnetic or non-magnetic phenotypes, which had supposedly excised the deletion target as suggested by PCR, but lost their insensitivity against kanamycin, indicating that parts of the suicide vector harboring the Km^r^ marker were likely still maintained in the genome. In our previous study, similar observations could be traced back to the inactivation of the *galK* gene encoding the lethal galactokinase, followed by spontaneous rearrangements in the absence of rigorous counterselection [53]. This explained our failure to enforce the proper deletion during counterselection in the presence of galactose, and in fact, the entire M04 region was still present in the genome (see Additional file 1: Fig. S3 for detail). To circumvent this problem, we separately deleted the essential *mamABop* first in strain ∆TZ-15, yielding strain ∆TZ-16, in which we attempted subsequent deletion of the residual M04 region. Several kanamycin sensitive (Km^s^), non-magnetic clones were obtained in this regime, which however again yielded diverse PCR products only roughly similar to the expected size spanning over the targeted excision site. Nevertheless, one of the clones (still tentatively termed ∆TZ-16), was selected as parent for later re-insertion of pMDJM3 harboring a compact version of the magnetosome biosynthesis gene clusters (see below).

In contrast to the troublesome M04 deletion, one-step deletion of the even larger region M13 in the background of ∆TZ-15 was obtained readily and yielded plenty of expected non-magnetic clones, in which the proper deletion of M13 could be confirmed by PCR spanning over the targeted excision site. This yielded strain ∆TZ-17. Like the respective single deletion mutants ∆M04 and ∆M13 in the WT background [53], both intermediate strains ∆TZ-16 and ∆TZ-17 showed conspicuous irregularly shaped electron dense particles (EDPs) between 10 and 125 nm in size in electron micrographs (see Fig. 6a below, white arrows), which were previously shown to be rich in potassium, phosphorus and oxygen, and to be unrelated to magnetosome biosynthesis [53].

In the final step, restoration of magnetosome biosynthesis was attempted in the two multiple deletion strains. This was achieved by insertion of pMDJM3 or pMDJM3-*gusA*, variants of pTpsMAG1 [60] harboring the compact set of *mam*/*mms/feo* genes and *lox* sites for restoration of antibiotic resistance to generate a marker-less mutant, and in case of pMDJM3-*gusA* in addition encoding the enzyme GusA (glucuronidase) as a chromogenic reporter. The *gusA* gene was added next to the *mamXYop* as entrapment for spontaneous mutations in a genetic stability assay to ∆TZ-16 and ∆TZ-17 (see below). As control, *gusA* was also inserted into the WT strain at the same genomic position next to the *mamXYop* as in pMDJM3. The region downstream of *mamXYop* was chosen as site for *gusA* insertion, since spontaneous deletions, insertions and rearrangements of this particular region were observed repeatedly as a virtual hotspot during routine genetic manipulation (unpublished observations). This is possibly caused by its close proximity (~ 11.4 kb) to the two *tn-tandem* copies described above, and often accompanied by impaired magnetosome phenotypes akin a *mamXYop* deletion [61]. Thus, the strains ∆TZ-16::MAG-*gusA*, ∆TZ-17::MAG, ∆TZ-17::MAG-*gusA* and WT-*gusA* (Fig. 4) were generated.

### Genome analysis of final multiple mutant strains

**Fig. 5:**
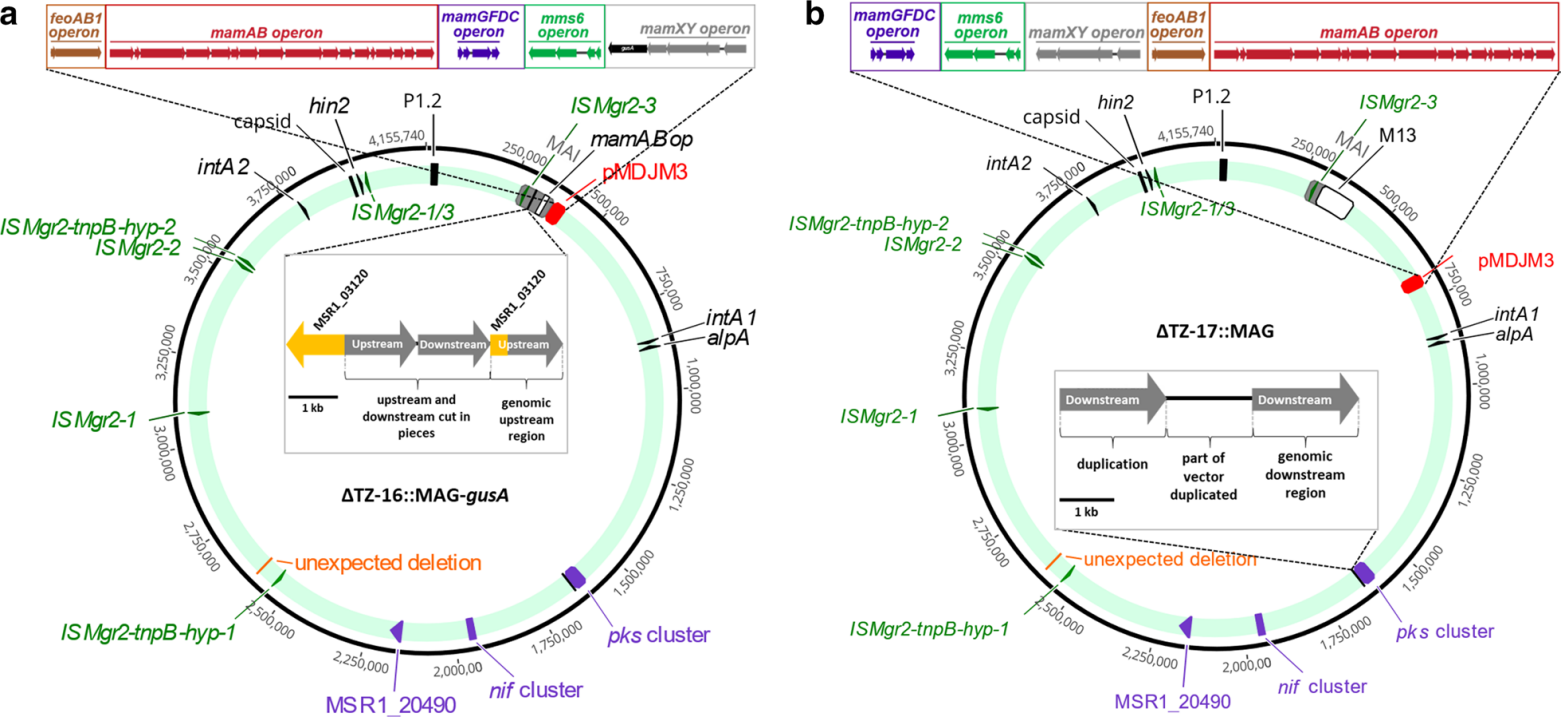
Schematic presentation showing an overview of ∆TZ-16::MAG-*gusA* (**a**) and ∆TZ-17::MAG (**b**) genotypes. Green circle shows the final genotype including unexpected insertions or deletions by resequencing of strains ∆TZ-16::MAG-*gusA* and ∆TZ-17::MAG. Grey: MAI; white: M13 or *mamABop* deletions; black arrows: parts of predicted prophage sets, integrase and excisionase genes; green: most active insertion element genes; purple: irrelevant gene cluster. Enlargements indicate unexpected duplication of vector remnants. In ∆TZ-16::MAG-*gusA* (**a**) remnants and duplications of up- and downstream regions of *pORFM-GalK-M04* suicide vector are still located within *msr1_03120* (encoding a putative secreted effector protein PipB) at the position (305,858 nt) targeted for deletion. As observed before, parts of up- and downstream homologous regions were found fragmented and duplicated. pMDJM3-*gusA* of strain ∆TZ-16::MAG-*gusA* has been inserted within the intergenic region between *mamY* (*msr1_03880*) and the adjacent transposon gene (*msr1_03890*) (red box). **b** pMDJM3 in strain ∆TZ-17::MAG is located at nt position 699,709 (red box) within the *ruvB* gene (*msr1_07040*) encoding a putative holliday junction ATP-dependent DNA helicase. Remnants and duplication of *pORFM-GalK-pks* suicide vector are located at nt position 1,646,447 (intergenic region upstream of *msr1_15660*) in strains ∆TZ-16::MAG-*gusA* and ∆TZ-17::MAG. Additionally, an unintended spontaneous 178 bp deletion in *fhaB 2* gene (*msr1_24320*) encoding a filamentous hemagglutinin is present at nt position 2,599,005 (green circle, orange), and an insertion of a copy of *ISMgr2* at nt position 3,961,873 into *cph1 40* gene (*msr1_37870*) encoding a phytochrome-like protein was found in both strains (green circle, dark green)

To verify the multiple introduced deletions, as well as possible unintended mutations and rearrangements that might have occurred during the numerous rounds of manipulation, at this point the two final multiple mutant strains ∆TZ-16::MAG-*gusA* and ∆TZ-17::MAG were subjected to genome resequencing. In strain ∆TZ-16::MAG-gusA this revealed that the region M04, which we attempted to delete in the last step, was still present as already suspected, except for *mamABop*, which had been removed already in the previous step in ∆TZ-16. As a consequence, ∆TZ-16::MAG-*gusA* is merodiploid for all magnetosome operons but *mamABop* and *feoAB1op*. All introduced *mam*/*mms/feo* genes were found to be present next to endogenous *mamXYop* (Fig. 5a, red box), although some with silent or neutral point mutations. However, conspicuously, the order of the introduced operons (*feoAB1-mamAB-mamGFDC-mms6-gusA-mamXY*) was shuffled compared to their original order on pMDJM3-*gusA* (*mamAB-feoAB1-mamXY-gusA-mms6-mamGFDC*). Apart from the failed M04 deletion, all other deletions introduced into ∆TZ-16::MAG-*gusA* were exactly as intended. However, besides a number of point mutations, a few larger indel mutations were found in genome regions likely to be irrelevant for magnetosome biosynthesis. These include the *pORFM-GalK-M04* suicide vector within *msr1_03120* (nt position 305,858), a 178 bp spontaneous deletion at nt position 2,599,005 in *msr1_24320* (encoding a filamentous hemagglutinin) and an insertion of a copy of *ISMgr2* at nt position 3,961,873 (with *msr1_37870* encoding a phytochrome-like protein).

In ∆TZ-17::MAG the entire M13 region was confirmed to be deleted exactly as intended (Fig. 5b, green circle). pMDJM3 was inserted at nt position 699,709 (Fig. 5b, green circle, red box). All introduced *mam*/*mms/feo* genes were found to be identical to pMDJM3 with respect to sequences and order. In addition to the successful M13 deletion, also all other introduced deletions were exactly as intended. A short remnant (3 558 bp) of suicide vector *pORFM-GalK-pks* was found inserted at nt position 1,646,447 (considered to be neutral), showing a duplication of the downstream homologous region like in ∆TZ-16::MAG-*gusA*. Again, the same spontaneous indel mutations in other chromosomal regions as in strain ∆TZ-16::MAG-*gusA* were also present in ∆TZ-17::MAG, indicating that these mutations had occurred already at an earlier stage of mutagenesis.

### Phenotypic characterization of ∆TZ-16::MAG-*gusA* and ∆TZ-17::MAG-*gusA*

#### Growth characteristics and magnetosome biomineralization

**Fig. 6:**
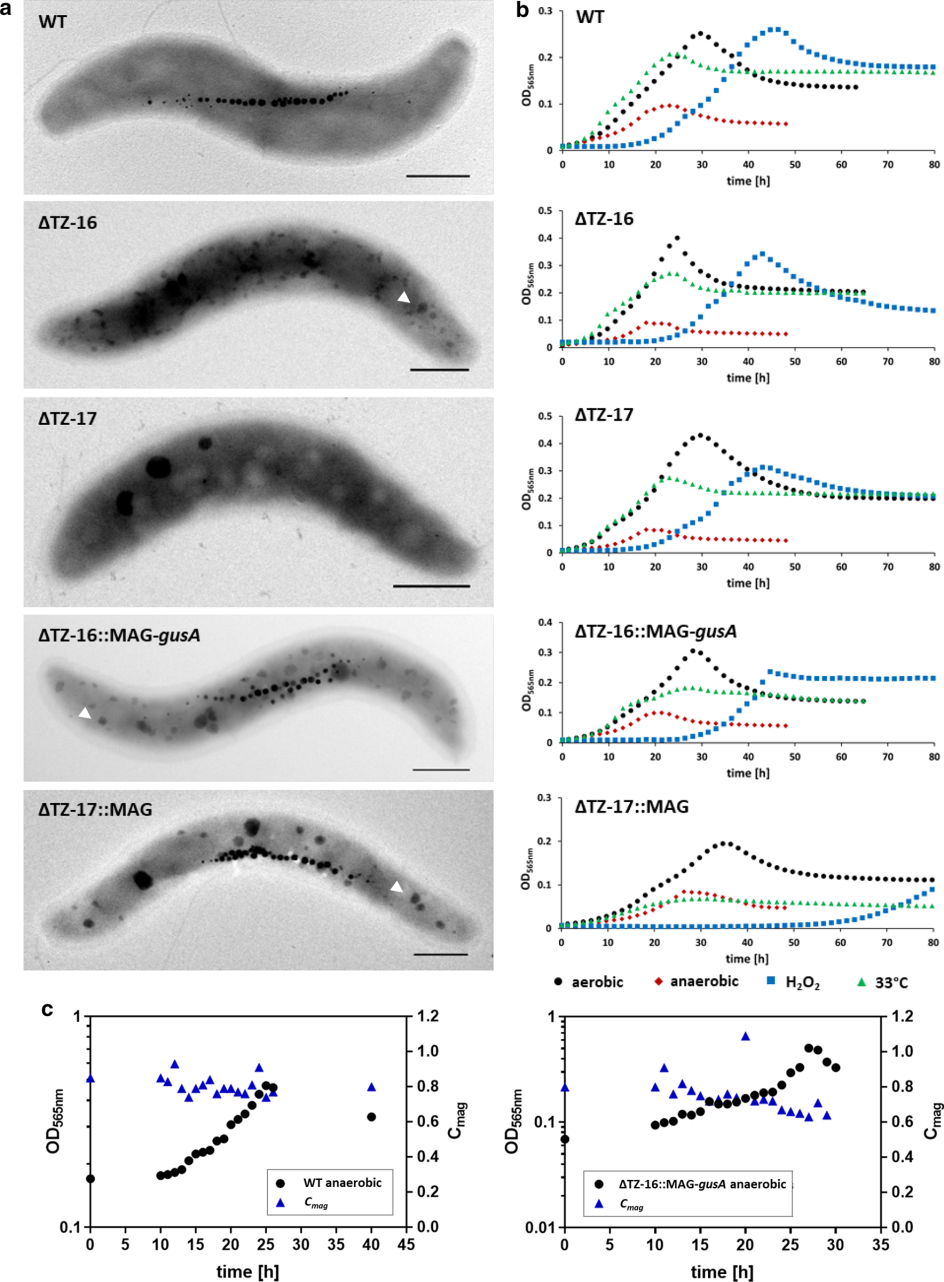
Phenotypic characterization of multiple deletion mutants. **a** Electron micrographs of the non-magnetic pre-'chassis' strains ∆TZ-16 and ∆TZ-17 and the final complemented prospective chassis ∆TZ-16::MAG-*gusA* and ∆TZ-17::MAG-*gusA* (scale bars 500 nm), white arrows indicate EDPs and **b** cell growth under aerobic and anaerobic conditions as well as oxidative stress (H_2_O_2_) and heat stress (33 °C). Each strain was analyzed in triplicates and the curves show the average while standard deviation was below 5%. **c** Growth curves and *C*_*mag*_ (i.e., a proxy for the average magnetic orientation of bacterial cells in liquid media based on light-scattering [63]) of WT strain and ∆TZ-16::MAG-*gusA* during anaerobic fermentation

Complementation of non-magnetic ∆TZ-16 (lacking 3.489% of the WT genome) and ∆TZ-17 (lacking 5.477%) with pMDJM3 restored the formation of WT-like magnetosome numbers and sizes, and cells had electron dense particles (EDP) (Fig. 6a, white arrows), similar as observed before in the corresponding single deletion strains of the eliminated parts of the MAI [53]. Microplate-scale experiments with strains ∆TZ-16::MAG-*gusA* and ∆TZ-17::MAG-*gusA* under aerobic and anaerobic nitrate-reducing conditions indicated WT-like or slightly delayed cell growth compared to WT (Fig. 6b). To analyze growth at higher cell densities, strains ∆TZ-16::MAG-*gusA* and ∆TZ-17::MAG-*gusA* were in addition cultivated in a larger volume (3 l) in a bioreactor under controlled anaerobic conditions, which are known to be optimal for magnetosome biomineralization [28, 62]. Figure 6c shows exemplary results for strain ∆TZ-16::MAG-*gusA* compared to the WT. Both strains reached a final OD of > 0.5, compared to only ca. 0.1 typically observed in microplate growth. Again, growth of strains ∆TZ-16::MAG-*gusA* and ∆TZ-17::MAG-*gusA* was WT-like, indicating that loss of the eliminated genes was neutral for growth under controlled conditions. Strains ∆TZ-16::MAG-*gusA* and ∆TZ-17::MAG were also tested regarding their growth performance after challenging them with the antibiotic MMC. Similar as the single deletion strain of the putative phage integrase gene *hin2,* both strains survived concentrations up to 0.3 µg/ml MMC, while WT was entirely inhibited at 0.3 µg/ml MMC (Additional file 1: Fig. S4).

#### Stability of the reporter gene gusA and the magnetic phenotype

To analyze whether the combined multiple deletion of IS elements in *M. gryphiswaldense* affects the incidence of spontaneous mutations, we employed an assay to estimate the genetic stability of expressed foreign genes as well as the stability of the magnetic phenotype using the reporter gene *gusA* as a 'trap' (Additional file 1: Fig. S5A), similar as reported for other marker genes in different bacteria [64]. Mutational inactivation of *gusA* causes the loss of the ability to cleave X-Gluc into blue dye, hence resulting in white (magnetosome-free) or brownish (magnetosome forming) colonies (Additional file 1: Fig. S5). After ten sequential passages under aerobic conditions (alternating between 4 h at 4 °C and 44 h at 28 °C, conditions which were previously found to favor spontaneous mutations [41], 12 independent parallels (equivalent to ~ 4.2 * 10^3^ cells for each strain and time point) were plated and visually screened. Out of ~ 2.5 * 10^4^ cells in total, 2–3% of colonies had lost their blue color. Overall, among the about 7.1 * 10^2^ white clones, we analyzed 192 white clones of each WT-*gusA*, ∆TZ-16::MAG-*gusA* and ∆TZ-17::MAG-*gusA* in which *gusA* was found to be inactivated by different types of mutations, including point mutations (80.6%), insertions (19.1%) and deletions (0.3%). The majority of the point mutations observed were base deletions (40%) and insertions (50%) causing frame shifts, while base substitutions represented the minority (10%). The types of point mutation were independent from time point or strain, and most mutations were found within a range of 500 bp of the *gusA* gene encoding the catalytic center of the GusA enzyme [65]. Furthermore, deletions of 62 bp of the *gusA* gene were found within the 25% N-terminal portion of the GusA protein.

All larger *gusA* insertions were found to belong exclusively to the two types of IS elements that we had already identified in our preliminary experiments described above [*ISMgr2* (7.3%) and *tn-tandem* (92.7%)], present both in cells before (t0) and after (t10) passaging. The high frequency observed for *tn-tandem* insertions might have been probably caused by its high abundance or close proximity (~ 11.4 kb) of a copy to the *gusA* reporter. While the total number of mutations between time points t0 and t10 did not significantly vary (Table 1), the number of all insertions was substantially reduced by ca. 60–75% in ∆TZ-16::MAG-*gusA* and ∆TZ-17::MAG-*gusA* compared to WT-*gusA*, likely due to the successful elimination of several active IS elements described above.

**Table 1:** Overview of identified mutations in the reporter gene *gusA*

	Point mutations	Insertions		Deletions
		*Tn-tandem*	*ISMgr2* (TnpA/B)	
t0				
WT-*gusA*	64	32	–	–
∆TZ-16::MAG-*gusA*	90	6	–	–
∆TZ-17::MAG-*gusA*	87	9	–	–
t10				
WT-*gusA*	56	30	8	2
∆TZ-16::MAG-*gusA*	81	15	–	–
∆TZ-17::MAG-*gusA*	86	10	–	–

In addition to mutations in *gusA*, reduction or loss of the magnetic phenotype was found in a minority of clones from white or brown colonies which had lost their blue color. Reduced magnetic phenotypes displaying WT-like magnetite crystals flanked by flake-like particles could be observed in several of WT-*gusA* clones after ten passages (Additional file 1: Fig. S5B), which likely indicates a second mutation (i.e., in addition to the point mutations within *gusA*) in *mamXYop* [61]. Furthermore, the loss of the magnetic phenotype coincident with *gusA* inactivation could be observed in three clones of ∆TZ-16::MAG-*gusA* for time point t10. This could be the result of spontaneous homologous recombination between identical stretches of DNA in this partially merodiploidic strain.

## Discussion

In this study, we established an approach for large-scale combinatory genome reduction of the magnetotactic bacterium *M. gryphiswaldense*. By repeated circles of deletion, we generated a library of strains in which different multiple genomic segments were erased. These strains might each serve as different starting points in future genome streamlining approaches by recombination with further favorable deletions and insertions.

In total, we completed the combination of 16 single deletions from this and our previous work [53] into each of the two strains ∆TZ-16 and ∆TZ-17, in which in addition large parts of the fragmented MAI were functionally replaced by a compact version of the magnetosome biosynthesis gene clusters.

On average, one round of deletion typically took about 3 weeks, and after some technical streamlining, the 16 subsequent rounds could have been completed in about 12 months of work. Independent of the target size, all rounds of successful deletions were largely completed with similar efficiencies as for respective single deletions. An exception was the unsuccessful deletion of the M04 region which might be especially problematic due to the abundance of transposon genes close to the regions targeted for homologous recombination. Several undesired mutations and spontaneous rearrangements were found to have occurred during recursive deletions. This emphasizes the need of genome resequencing of key intermediates and final strains. Again, most of these spontaneous rearrangements were caused by either homologous or illegitimate recombination and could be traced back to the spontaneous inactivation of the lethal *galK* marker harbored on the suicide vectors for homologous recombination, thereby preventing effective counterselection of proper double-crossovers.

However, despite of these caveats, we succeeded in the construction of one final strain (∆TZ-17) with a genome reduction by non-overlapping ~ 227,600 bp, which is equivalent to about 5.5% of the entire genome. In this strain all targeted deletions and the reinsertion of the compacted magnetosome gene clusters were found to be exactly as intended, with only few minor spontaneous mutations in regions irrelevant for growth and magnetosome biosynthesis under laboratory conditions. This confirms that if used with caution, the method is sufficiently efficient and reliable for multiple genome editing.

In other bacteria, multiple genome reductions of different extents by similar approaches were previously reported. For example, one of the first studies in *E. coli* K12 resulted in a genome reduction up to 15% [47], and the genome of *E. coli* could be further shrunk by > 29%, changing cell size and nucleoid organization of engineered cells [66]. A “MiniBacillus” was constructed from *Bacillus subtilis*, in which a total of 42.3% was eliminated [67]. However, top-down genome reduction approaches in model organisms other than *E. coli* and *B. subtilis* have been more limited, and in some cases involved the combined, stepwise efforts of several labs [43]. For example, in *C. glutamicum* multiple approaches resulted first in the targeted deletion of 11 distinct regions with a total size of 250 kilobase pair (kbp) [68, 69], followed by an untargeted approach via insertion and excision [70]. In another random approach, 42 mutants in a range of 0.2–186 kb were generated, which revealed a total of 393.6 kb (11.9%) of the *C. glutamicum* R genome to be non-essential under standard laboratory conditions [70]. More recently, five of the 36 single large deletions identified by Unthan et al. [46] were later combined in a chassis strain of *C. glutamicum*, in which 13.4% of the genome were eliminated [45]. Similar approaches with *P. putida* resulted in genome reductions of 4.1% [71], 4.3% [48] and 4.12% [72], respectively, as well as in *Lactococcus lactis* (2.83% reduction) [73], *Streptomyces avermitilis* (1.4 Mb reduction) [74, 75], while in *S. chattanoogensis* 1.3 Mb and 0.7 Mb regions were eliminated [76]. With a comparable reduction by nearly 5.5% of the genome, our study represents the first proof-of-principle for the feasibility of similar targeted approaches in a magnetotactic bacterium.

While many of the genome streamlining approaches described above led to beneficial properties, such as improved growth and recombinant protein production, as well as robustness against several stresses [34, 45, 47, 50, 77], others resulted in negative effects, such as growth deficiencies, decreased resistance against antibiotics and under several stress conditions, and reduced transformation efficiencies [45, 78]. In our study, most deletions were neutral with respect to magnetosome biosynthesis and growth. For example, our preliminary analysis suggested that neither the conjugation efficiency with replicative and insertional plasmids, nor the weak latent propensity of spontaneous cell lysis was affected in strains ∆TZ-16::MAG-*gusA* and ∆TZ-17::MAG-*gusA* (data not shown). As observed in our previous study, deletion of *mamABop* in ∆TZ-16 and ∆TZ-17 resulted in a growth advantage which became lost after 're-magnetization' by complementation. Among the several putative prophage genes of *M. gryphiswaldense*, only deletion of the recombinase gene *hin2* from P6 had an effect and resulted in a slightly improved resilience to mitomycin c (MMC)-induced stress in the final strains. This provides an indirect hint that P6 may be an active prophage, whose excision might be induced by MMC in WT cells, but further work, such as the identification of phage particles, will be necessary to confirm this. However, the combined deletion of other phage-related genes did neither further increase MMC resistance, nor generally enhance growth. On the contrary, deletion of the M13 region and prophage genes slightly impaired growth in the presence of oxidative stress, possibly for similar reasons as suggested by Wang et al. [78], who found that the presence of cryptic prophages may help bacteria to cope with adverse conditions and provide multiple benefits. For comparison, ∆TZ-16 lacks the ∆M13 deletion and therefore could be useful as an alternative parent strain with improved growth characteristics in follow-up genome streamlining studies.

From the ~ 120 transposable elements predicted in the 4.155 Mbp genome of *M. gryphiswaldense*, 30 are encoded within the ca. 100 kb MAI, and nine in addition in its ~ 33 kb adjacent region [1, 53]. However, our systematic approach revealed that only a minority of them, belonging to two families, seems to be responsible for the majority of spontaneous insertions. We detected an increased stability of the reporter *gusA* in both final multiple deletion strains, which was likely a result of the successful elimination of all *ISMgr2* elements described above, including one from the MAI. Future approaches should also aim for the removal of *ISMgr2-1*, *ISMgr2-2* and *ISMgr2-3* which might further decrease the rate of spontaneous mutations. However, deletion of multiple copies of the *tn-tandem*, the second group of identified troublemakers, or even the generation of a chassis stripped of all copies as accomplished in several other bacteria [51, 52] is currently not within realistic reach, due to the numerous abundance and extensive sequence similarity between multiple copies of IS elements, as well as their persistent tendency to spread during genetic manipulation.

## Conclusion

Overall, in this study we succeeded in further domestication and large-scale engineering of magnetotactic bacteria and showed the potential of combining multiple scarless deletions with high precision. We also generated a library of deletions, which represent building blocks for recombination with favorable deletions and insertions that can be used for the construction of improved 'chassis' strains in the future. Ultimately, this may turn *M. gryphiswaldense* into a versatile platform and microbial cell factory for synthetic biology and magnetosome production.

## Methods

### Bacterial strains, vectors, and cultivation conditions

Bacterial strains and plasmids used in this work are listed in Additional file 1: Table S1. *E. coli* strains were grown as previously reported [79]. *E. coli* WM3064 strains were grown in lysogeny broth (LB) medium supplemented with 25 µg/ml (final concentration) kanamycin (Km), and 1 mM dl-α,ε-diaminopimelic acid (DAP) at 37 °C. Liquid cultures of *M. gryphiswaldense* strains were grown microaerobically in flask standard medium (FSM) [28] at 28 °C under moderate shaking (120 rpm). Strains carrying the suicide or complementation plasmid were cultivated by adding 5 µg/ml Km. For cultivation on solid LB medium or FSM, 1.5% (w/v) agar was added. Cultivation from single *M. gryphiswaldense* colonies was conducted by transferring cell material into 150 µl FSM in 96-deep-well-plates (Eppendorf, Hamburg, Germany), prior to gradually increasing the culture volume. Optical density (OD) and magnetic response (*C*_*mag*_, i.e., a proxy for the average magnetic orientation of bacterial cells in liquid media based on light-scattering) of cells in the exponential growth phase were measured photometrically at 565 nm as previously described [63].

Growth experiments of *M. gryphiswaldense* were performed by using pre-cultures grown for two daily passages under microaerobic conditions at 28 °C. Cultures were adjusted to an initial OD of 0.01 and grown in a microplate reader (Tecan) under aerobic conditions at 28 °C or moderate heat stress at 33 °C. For induction of oxidative stress, 20 µM H_2_O_2_ were added prior to starting the growth experiments.

For cell growth after mitomycin C (MMC) induction, pre-cultures were adjusted to an initial OD of 0.08 and treated with MMC concentrations of 0.1–0.3 µg/ml for 8 h. Non-induced strains (0 µg/ml) served as controls. Then cultures were washed twice in FSM, and an initial OD of 0.01 was used to start growth experiments in the microplate reader under aerobic conditions at 28 °C.

In preliminary experiments, conditions could be defined (i.e. incubation with 0.2 µg/ml MMC for 8 h) in which growth of the WT was already somewhat impaired, yet still reached substantial yields (final OD of ca. 0.3), while slightly increased MMC concentrations (0.3 µg/ml) entirely abolished growth. Therefore, we used 8 h and 0.2–0.3 µg/ml MMC as efficient incubation conditions to analyze survival of mutants compared to the WT strain.

For cultivation in the fermenter, modified FSM was used adding 10 mM NaNO_3_ instead of 4 mM NaNO_3_ as alternative electron acceptor under anaerobic conditions. Growth experiments of WT and final strains were performed in 3 l BioFlow® 320 reactors (Eppendorf Bioprocess) equipped for the automatic control of pH (with H_2_SO_4_ or KOH), temperature, agitation, and nitrogen concentration. Data were directly saved at unit or in BioCommand software. Seed train was prepared 56 h before inoculation in falcon tubes and scaled up to 1 l flasks under anaerobic conditions.

### Molecular and genetic techniques

Oligonucleotides used as primers for amplification of DNA fragments were inferred from the working draft genome sequence of *M. gryphiswaldense* (GenBank accession number CP027526) [80] and purchased from Sigma-Aldrich (Steinheim, Germany). Construction of plasmids was performed by standard recombinant techniques as described in Zwiener et al. [53]. Generated constructs were sequenced by Macrogen Europe (Amsterdam, Netherlands) and sequence data analyzed with Geneious 8.0.5 (Biomatters Ltd).

#### Construction of markerless gene deletion vectors and mutants

Generation of single and multiple deletion mutants and WT-*gusA* insertion mutant was carried out by a tailored *galK* counterselection system as previously reported [53, 81].

We used chromosomal insertion and expression of magnetosome biosynthetic gene clusters, since previous work has shown that episomal expression in *M. gryphiswaldense* resulted in instability and inhomogenous expression of foreign and magnetosome genes [82, 83]. Multiple deletion mutants were complemented with the pMDJM3 cassette, a recyclable variant of pTpsMAG1 [60] containing *lox* sites next to the antibiotic marker and all operons necessary for magnetosome formation. For insertion of the chromogenic marker *gusA* into pMDJM3, RedET recombineering [84] was performed according to BAC Subcloning Kit (Gene Bridges) technical protocols.

### Analytical methods

#### Analysis of putative prophages

Analysis of putative prophages was performed by the phage search tool PHAST [55]. In PHAST, a prophage-like element was considered incomplete if its completeness score was less than 60, questionable if the score was in the range between 60 and 90, and complete if the score was above 90.

#### Re-sequencing of genomic DNA

Genomic DNA was isolated following the manual instructions of Quick-DNA Midiprep Plus Kit (Zymo Research Europe GmbH). For each isolated gDNA, two sequencing libraries were arranged, one for sequencing on the MiSeq platform (Illumina Inc, NL), and the second for sequencing on the GridION platform [Oxford Nanopore Technologies (ONT), UK]. The former was established using the TruSeq DNA PCR-free Library Kit (Illumina Inc., The Netherlands) and was carried out in a 2 × 300 nt run using a 600 cycle MiSeq Reagent Kit v3 (Illumina Inc, The Netherlands). For ONT sequencing, the Ligation Sequencing Kit SQK-LSK109 was used to arrange the libraries, which were in turn run on a R9.4.1 flow cell. Basecalling of the raw ONT data was carried out with guppy v3.2.8 [85]. For assembly, three assemblers were used: the canu assembler v1.8 [86] was utilized to assemble the ONT data. The resulting assembled contigs were polished applying first the ONT data with racon v1.3.3 [87] and medaka v0.11.5 (Oxford Nanopore Technologies), both relying on minimap2 v2.17-r943 [88] for mapping, followed by switching to the Illumina data and the pilon polisher v1.22 [89] for a total of 10 rounds. For the first 5 rounds, bwa mem [90] was utilized as a mapper, for the final 5 cycles, bowtie2 [91] was applied. In addition, the Illumina data was assembled using newbler v2.8 [92] and both data sets were gathered using unicycler [93]. All assemblies were compared with each other and examined for synteny using r2cat [94]. All three assemblies were combined and manually curated using consed [95]. Annotation of the finished genomes was carried out using prokka v1.11 [96] SNPs and small indels were identified using snippy v4.0 [97] while larger rearrangements were recognized manually using SnapGene (GSL Biotech).

#### Preparation of samples for transmission electron microscopy (TEM)

For conventional transmission electron microscopy (TEM) of cell and magnetosome morphologies, cultures were grown under microoxic conditions in FSM at 28 °C. Overnight cultures were fixed in 1.5% formaldehyde and adsorbed onto carbon-coated copper-mesh grids (Science Services, Munich, Germany). TEM was performed on a JEOL 1400 (Japan) with an acceleration voltage of 80 kV and micrographs were analyzed using the software ImageJ [98].

#### Genetic stability assay

To test genetic stability of the reporter *gusA*, overnight cultures were transferred to 96-well-plates and incubated for ten passages under aerobic conditions alternating between 4 h at 4 °C and 44 h at 28 °C. 12 independent parallels of each strain were plated on FSM agar moistened with 250 µl of a 10 mg/ml X-Gluc stock solution on its surface. Clones producing active GusA could be visually screened by their blue color, while mutations inside *gusA* resulted in the loss of the ability to cleave X-Gluc, and thus in in white or brownish colonies after 7–10 days of incubation at 28 °C. Colonies were counted at time points 0 (t0) and after ten passages (t10) and mutations identified by PCR and sequencing.

## Supplementary Information

**Additional file 1 of “Towards a ‘chassis’ for bacterial magnetosome biosynthesis: genome streamlining of** ***Magnetospirillum gryphiswaldense*** **by multiple deletions”: Table S1.** Overview of all single deletion mutants which were also combined in strains ∆TZ-16 and ∆TZ-17. **Table S2.** Overview of primers used in this study. UF = upstream forward; UR = upstream reverse; DF = downstream forward; DR = downstream reverse. **Figure S1.** Molecular organization of *nif* operon in *M. gryphiswaldense*. The deleted nitrogen fixation cluster comprises 16 genes necessary for nitrogen fixation (shown in red): *nifWABZTHDK*, *fixABC*, *draGT* and three ferredoxins (MSR1_18560; MSR1_18600; MSR1_18640). Black arrows represent other genes encoding a putative rubrerythrin protein (MSR1_18580), a SIR2-like domain containing protein (MSR1_18630), a GAF domain-containing protein (MSR1_18650), a biliverdin-producing heme oxygenase (MSR1_18660) and a tRNA (MSR1_18670). **Figure S2.** Phenotypic characterization of multiple deletion mutants. Electron micrographs of combinatorial deletion mutants ∆TZ-01–∆TZ-15. Scale bars: left columns 500 nm; right columns 100 nm. Cell growth of strains ∆TZ-01–∆TZ-15 under aerobic and anaerobic conditions as well as oxidative stress (H_2_O_2_) and moderate heat stress (33 °C). Each strain was analyzed in triplicates and each curve shows the average. **Figure S3.** Genetic organization of the Km^r^, false positive mutant ∆TZ-15∆M04 K752 Km^r^. The targeted M04 had not been deleted but was still maintained in the genome. A large part (~ 9.1 kb) of the 10.2 kb deletion vector *pORFM-GalK-M04* harboring the *Km*^*r*^ gene was found to be inserted at the intended site, but harboring a spontaneous duplication of both the upstream and downstream homologous regions intended for targeted insertion of the deletion construct by homologous recombination. In addition, the *galK* gene was inactivated by insertion of a copy of the IS element *ISMgr2* into the central region. **Figure S4.** Growth profiles of these strains induced with MMC with concentrations between 0.2 and 0.3 µg/ml MMC, induced 8 h. Cells were washed twice, adjusted to initial OD and growth experiments started at 28 °C under aerobic conditions and each strain was analyzed in triplicates while each curve shows its average (standard deviation < 5%). **Figure S5.** Experimental procedure of the genetic stability assay (**A**) and identified magnetosome phenotypes (**B**). Blue arrows indicate blue colonies while white/brown colonies are marked by black arrows (**A**). TEM micrographs (**B**) show WT-like magnetosome chains (upper micrograph) and flake-like particles (lower micrograph, white arrows) could be observed in several of WT-*gusA* clones after ten passages. Scale bars: 100 nm.

## Data Availability

The datasets used and analyzed during the current study are available from the corresponding author on reasonable request.

## Abbreviations

MAI: Magnetosome Island
WT: Wild type
IS element: Insertion element
MNP: Magnetic nanoparticles
MMC: Mitomycin C
*tn-tandem*: Transposon-tandem
Km^r^: Kanamycin resistant
Km^s^: Kanamycin sensitive
EDP: Electron dense particles
X-Gluc: 5-Bromo-4-chloro-3-indolyl-β-d-glucuronide
DAP: dl-α,ε-Diaminopimelic acid
