# Supplementary material for: Towards a 'chassis' for bacterial magnetosome biosynthesis: genome streamlining of Magnetospirillum gryphiswaldense by multiple deletions
Source: Microb Cell Fact. 2021 Feb 4;20:35. doi: 10.1186/s12934-021-01517-2 (PMC7860042; doi:10.1186/s12934-021-01517-2)
Supplement: Supplementary file 1 — Additional file 1 of “Towards a ‘chassis’ for bacterial magnetosome biosynthesis: genome streamlining of Magnetospirillum gryphiswaldense by multiple deletions”: Table S1. Overview of all single deletion mutants which were also combined in strains ∆TZ-16 and ∆TZ-17. Table S2. Overview of primers used in this study. UF = upstream forward; UR = upstream reverse; DF = downstream forward; DR = downstream reverse. Figure S1. Molecular organization of nif operon in M. gryphiswaldense. The deleted nitrogen fixation cluster comprises 16 genes necessary for nitrogen fixation (shown in red): nifWABZTHDK, fixABC, draGT and three ferredoxins (MSR1_18560; MSR1_18600; MSR1_18640). Black arrows represent other genes encoding a putative rubrerythrin protein (MSR1_18580), a SIR2-like domain containing protein (MSR1_18630), a GAF domain-containing protein (MSR1_18650), a biliverdin-producing heme oxygenase (MSR1_18660) and a tRNA (MSR1_18670). Figure S2. Phenotypic characterization of multiple deletion mutants. Electron micrographs of combinatorial deletion mutants ∆TZ-01–∆TZ-15. Scale bars: left columns 500 nm; right columns 100 nm. Cell growth of strains ∆TZ-01–∆TZ-15 under aerobic and anaerobic conditions as well as oxidative stress (H2O2) and moderate heat stress (33 °C). Each strain was analyzed in triplicates and each curve shows the average. Figure S3. Genetic organization of the Kmr, false positive mutant ∆TZ-15∆M04 K752 Kmr. The targeted M04 had not been deleted but was still maintained in the genome. A large part (~ 9.1 kb) of the 10.2 kb deletion vector pORFM-GalK-M04 harboring the Kmr gene was found to be inserted at the intended site, but harboring a spontaneous duplication of both the upstream and downstream homologous regions intended for targeted insertion of the deletion construct by homologous recombination. In addition, the galK gene was inactivated by insertion of a copy of the IS element ISMgr2 into the central region. Figure S4. Growth profiles of these strains [file 12934_2021_1517_MOESM1_ESM.docx]

## Additional file 1

**Table S1. Overview of all single deletion mutants which were also combined in strains ∆TZ-16 and ∆TZ-17.**

| Name of single deletion mutant | Deleted genes | Total extent of deletion |
| --- | --- | --- |
| ∆M04 | MSR1_03150 to MSR1_03880 | 65,965 bp |
| ∆M13 | MSR1_03150 to MSR1_04210 | 98,984 bp |
| ∆*feoAB1op* | MSR1_02660 to MSR1_02670 | 2,406 bp |
| ∆*mamABop* | MSR1_03340 to MSR1_03500 | 16,364 bp |
| ∆P7 | MSR1_02780 to MSR1_02990 **not deletable in this study** | 12,793 bp |
| ∆*intA1* | MSR1_08690 | 1,284 bp |
| ∆*intA2* | MSR1_36030 | 1,226 bp |
| ∆*hin2* | MSR1_37790 | 1,333 bp |
| ∆*alpA* | MSR1_08890 | 255 bp |
| ∆P1.2 | MSR1_00080 to MSR1_00280 | 19,489 bp |
| ∆P1.3 | MSR1_00140 to MSR1_00280 | 12,826 bp |
| ∆capsid | MSR1_37460 to MSR1_37540 | 7,651 bp |
| ∆*pks* cluster | MSR1_15630 to MSR1_15650 | 39,049 bp |
| ∆*nif* cluster | MSR1_18530 to MSR1_18720 | 17,608 bp |
| ∆MSR1_20490 | MSR1_20490 | 31,026 bp |
| ∆*ISMgr2-1* | MSR1_29210 to MSR1_29220 | 1,244 bp |
| ∆*ISMgr2-2* | MSR1_33470 to MSR1_33480 | 1,304 bp |
| ∆*ISMgr2-3* | MSR1_02780 to MSR1_02790 | 1,434 bp |
| ∆*ISMgr2-tnpB-hyp-1* | MSR1_23730 to MSR1_23750 | 1,214 bp |
| ∆*ISMgr2-tnpB-hyp-2* | MSR1_33640 to MSR1_33660 | 1,990 bp |

**Table S2. Overview of primers used in this study. UF = upstream forward; UR = upstream reverse; DF = downstream forward; DR = downstream reverse.**

| Primer | Sequence (5’ to 3’) | Description |
| --- | --- | --- |
| TZ199 | AACTCTCCAGCGCTTCGGCAAAAG | ∆M04 UF |
| TZ200 | CAGACGGATCGCTTCTCGCCGAACATAAGGGCTGCTCCCGTGG | ∆M04 UR |
| TZ201 | CCACGGGAGCAGCCCTTATGTTCGGCGAGAAGCGATCCGTCTG | ∆M04 DF |
| TZ202 | GCGCCCATCAGCTTGGCCC | ∆M04 DR |
| TZ515 | CACAGCGCAGCGCATTG | ∆M13 UF |
| TZ516 | TTGCGTGACTGTTAACTAAACAACGTTAAGGTGGACTTG | ∆M13 UR |
| TZ517 | TAGTTAACAGTCACGCAACGGCGC | ∆M13 DF |
| TZ509 | GGTGGTGGGCACGCC | ∆M13 DR |
| RU423 | GGAGGAGTTCCAGGGTCTTGTTCTGGTGGCGGAGCG | ∆*feoAB1op* UF |
| RU424 | CAGCAGCTGCGCCATGCTGTCCG | ∆*feoAB1op* UR |
| RU425 | GCCTTGCCAGCTGCGCATGC | ∆*feoAB1op* DF |
| RU426 | CACCAGAACAAGACCCTGGAACTCCTCCTTAT | ∆*feoAB1op* DR |
| TZ049 | AGCTAACAAACAACGCGCTGCCTC | ∆*mamABop* UF |
| TZ196 | GCGCTCCGGGCAGAATGTCGCTGGGACGCTGGCTCGGC | ∆*mamABop* UR |
| TZ197 | GCCGAGCCAGCGTCCCAGCGACATTCTGCCCGGAGCGC | ∆*mamABop* DF |
| TZ198 | ATCCGACCGGGCGGCCTC | ∆*mamABop* DR |
| TZ435 | GATGGTACCTAGGATGAGGGTCATGGCC | ∆P7 UF |
| TZ436 | ATGAAGCCTTTGCCCGTGAATCCTGCCGTT | ∆P7 UR |
| TZ437 | CGGCAGGATTCACGGGCAAAGGCTTCATCG | ∆P7 DF |
| TZ438 | AGCGGCCGCATCGATCGCGTTCTGCTCTCG | ∆P7 DR |
| TZ425 | GGTTTTCGTGAATATAATTTTTCAGCC | ∆*intA1* UF |
| TZ426 | TAGCCCCCCCGAGCGAGCCCCCGA | ∆*intA1* UR |
| TZ427 | GGGCTCGCTCGGGGGGGCTATGCCC | ∆*intA1* DF |
| TZ428 | GGATACGCCTCCAGGCTTC | ∆*intA1* DR |
| TZ430 | CAGTCTGGAACGGCGC | ∆*intA2* UF |
| TZ431 | TCCCCGGCGGGGCCGAATCTCCATGGG | ∆*intA2* UR |
| TZ432 | AGATTCGGCCCCGCCGGGGAAAAATATT | ∆*intA2* DF |
| TZ433 | GTCTTATTCCCGATCTTGACCG | ∆*intA2* DR |
| TZ666 | CCGCCGCATCCACGATGAG | ∆*hin2* UF |
| TZ667 | GGCGGTCATTCATCGGCTGGTCTCCAACTTGC | ∆*hin2* UR |
| TZ668 | AGCCGATGAATGACCGCCGACATCCGG | ∆*hin2* DF |
| TZ669 | CGATCCCGCAGCAGAAAGGTATG | ∆*hin2* DR |
| TZ448 | TGGTACCTAGGATAAATAGCCTCCCCGACACG | ∆*alpA* UF |
| TZ449 | AAAGGGAGAAATCCGGGCCATCCCCGTTT | ∆*alpA* UR |
| TZ450 | AACGGGGATGGCCCGGATTTCTCCCTTTCG | ∆*alpA* DF |
| TZ451 | CGGCCGCATCGATCCCTCTCTCCAGCATCTTGC | ∆*alpA* DR |
| TZ531 | AACCGGCCAACACGCC | ∆P1.2 UF |
| TZ457 | GGGCTGTTCAGCTTTTCAAGGGCTTAGCG | ∆P1.2 UR |
| TZ458 | TGAAAAGCTGAACAGCCCTGATAGGTCAGG | ∆P1.2 DF |
| TZ532 | CGCGTGGCCTTGAAACC | ∆P1.2 DR |
| TZ460 | TGCAGTAGGTCGACGATGGCAGCTTGCGCATACCGTTC | ∆P1.3 UF |
| TZ461 | GGGCTGTTCCCGTTAGCGTTGAACAGCC | ∆P1.3 UR |
| TZ462 | CGCTAACGGGAACAGCCCTGATAGGTCAGG | ∆P1.3 DF |
| TZ463 | GCTTGCGGCAGCGTGAAGCGCGTGGCCTTGAAACC | ∆P1.3 DR |
| TZ671 | CGAGACGTGGGCGTCCG | ∆capsid UF |
| TZ672 | ATGAACACGCGAGTGTGTCCATGATCGGGG | ∆capsid UR |
| TZ673 | ACACACTCGCGTGTTCATCTGGCAGAACGTTCAC | ∆capsid DF |
| TZ674 | TGTCAAATAACTCTTACCGTCAGGCC | ∆capsid DR |
| TZ628 | ACGCCGCCTTGTTCG | ∆*pks* cluster UF |
| TZ629 | CTGGATCGGGCTCATGCGCGCTCTC | ∆*pks* cluster UR |
| TZ630 | CGCATGAGCCCGATCCAGCATAATATGCGC | ∆*pks* cluster DF |
| TZ631 | GACCGCATAGGCCAAGACC | ∆*pks* cluster DR |
| TZ232 | GCGACGAGGCGAAGAAACTGGC | ∆*msr1_20490* UF |
| TZ233 | TTCCCCGAAGTCAGATTCTATGCCGCCCGCCGTGAATTTA | ∆*msr1_20490* UR |
| TZ234 | GCGGGCGGCATAGAATCTGACTTCGGGGAAAAGTACACTGAG | ∆*msr1_20490* DF |
| TZ235 | CGCCGCCGCATCGAGGAATTG | ∆*msr1_20490* DR |
| TZ564 | TTTGTGTTCATTCGTGTCGCC | ∆*ISMgr2-1* UF |
| TZ565 | GCCCCCTAAAATTCATTGCCATGCGCCAA | ∆*ISMgr2-1* UR |
| TZ566 | CAATGAATTTTAGGGGGCTGAAACAGAGC | ∆*ISMgr2-1* DF |
| TZ567 | GCCGACAACAGGTTGCCA | ∆*ISMgr2-1* DR |
| TZ569 | TTCTAAAATTTGCTGTGGTGCCC | ∆*ISMgr2-2* UF |
| TZ685 | GCCCCCTAAATGAGGAACGTCCCGCTTC | ∆*ISMgr2-2* UR |
| TZ686 | GTTCCTCATTTAGGGGGCTGAAACACTCATTG | ∆*ISMgr2-2* DF |
| TZ687 | TCATCCGGTCTTATGGAACGAAAG | ∆*ISMgr2-2* DR |
| TZ618 | AAATAGCTTCGAGACATACCCCC | ∆*ISMgr2-3* UF |
| TZ619 | CCCCTAAAATGCCCAATGGGCGAGA | ∆*ISMgr2-3* UR |
| TZ620 | CATTGGGCATTTTAGGGGGCTACAACACAAAG | ∆*ISMgr2-3* DF |
| TZ621 | TCAGACATGGCCCTCGG | ∆*ISMgr2-3* DR |
| TZ662 | GTGGAAAAACGGGCCCCCG | ∆*ISMgr2-tnpB-hyp-1* UF |
| TZ663 | CCGCATGGGTCAGATCGGCGCATCCTTACCG | ∆*ISMgr2-tnpB-hyp-1* UR |
| TZ664 | CCGATCTGACCCATGCGGAACACTGCGC | ∆*ISMgr2-tnpB-hyp-1* DF |
| TZ665 | GCGATTTCAGCCAGCGTTGATTG | ∆*ISMgr2-tnpB-hyp-1* DR |
| TZ608 | CATCCCGGAAATCAGCCAG | ∆*ISMgr2-tnpB-hyp-2* UF |
| TZ609 | CTTAGCCTCATCCACCCCACACATCTAATACACC | ∆*ISMgr2-tnpB-hyp-2* UR |
| TZ610 | GGGGTGGATGAGGCTAAGATCTCCGCACG | ∆*ISMgr2-tnpB-hyp-2* DF |
| TZ611 | ATGTAATCGCAATAGGCCGC | ∆*ISMgr2-tnpB-hyp-2* DR |


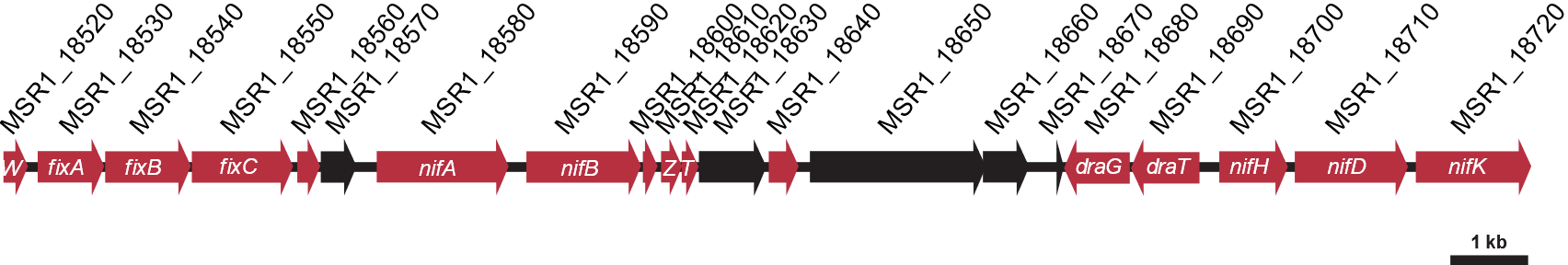


**Figure S1. Molecular organization of *nif* operon in *M. gryphiswaldense*.** The deleted nitrogen fixation cluster comprises 16 genes necessary for nitrogen fixation (shown in red): *nifWABZTHDK*, *fixABC*, *draGT* and three ferredoxins (MSR1_18560; MSR1_18600; MSR1_18640). Black arrows represent other genes encoding a putative rubrerythrin protein (MSR1_18580), a SIR2-like domain containing protein (MSR1_18630), a GAF domain-containing protein (MSR1_18650), a biliverdin-producing heme oxygenase (MSR1_18660) and a tRNA (MSR1_18670).


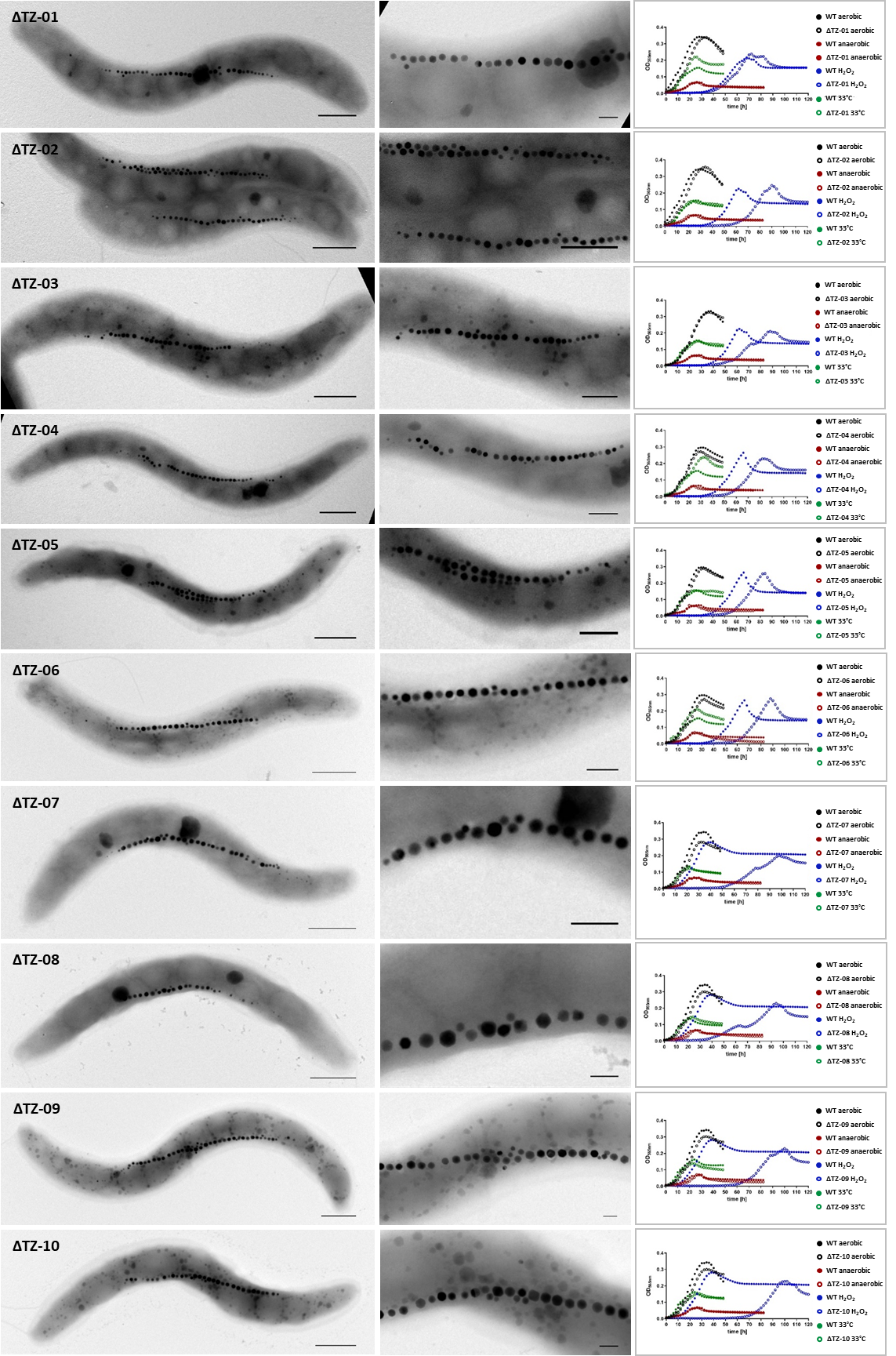


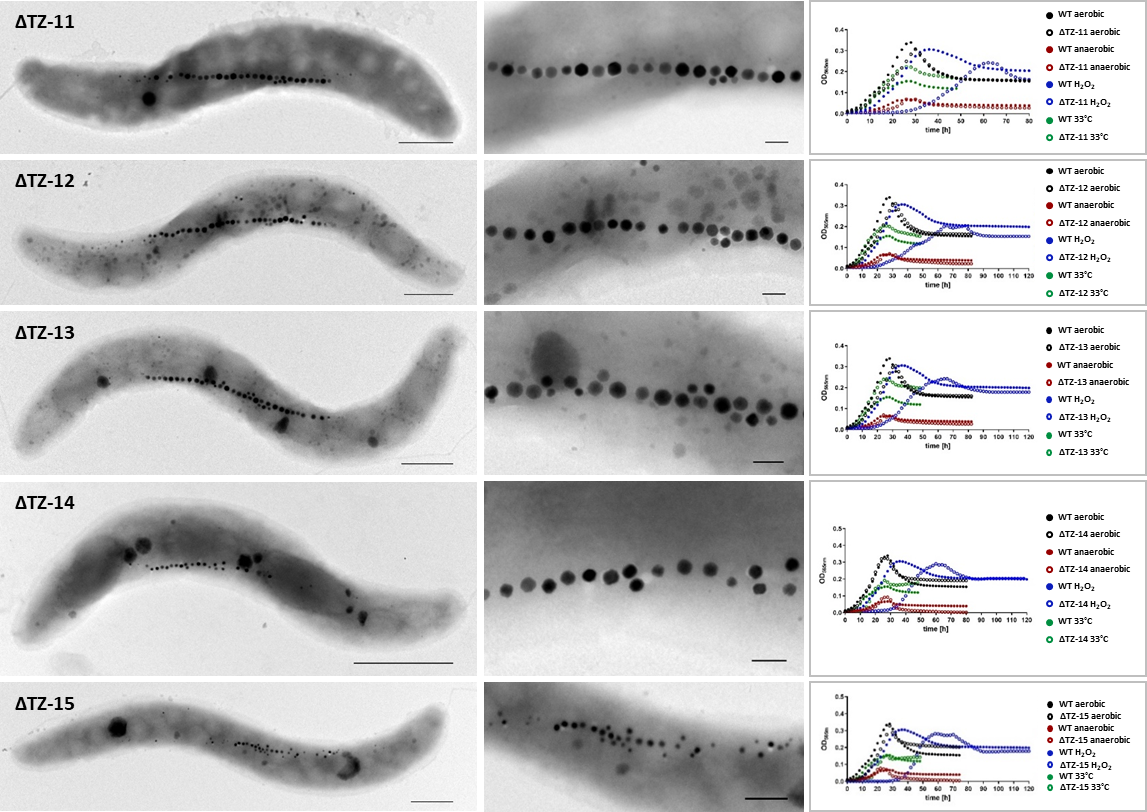


**Figure S2. Phenotypic characterization of multiple deletion mutants.** Electron micrographs of combinatorial deletion mutants ∆TZ-01–∆TZ-15. Scale bars: left columns 500 nm; right columns 100 nm. Cell growth of strains ∆TZ-01–∆TZ-15 under aerobic and anaerobic conditions as well as oxidative stress (H_2_O_2_) and moderate heat stress (33°C). Each strain was analyzed in triplicates and each curve shows the average.


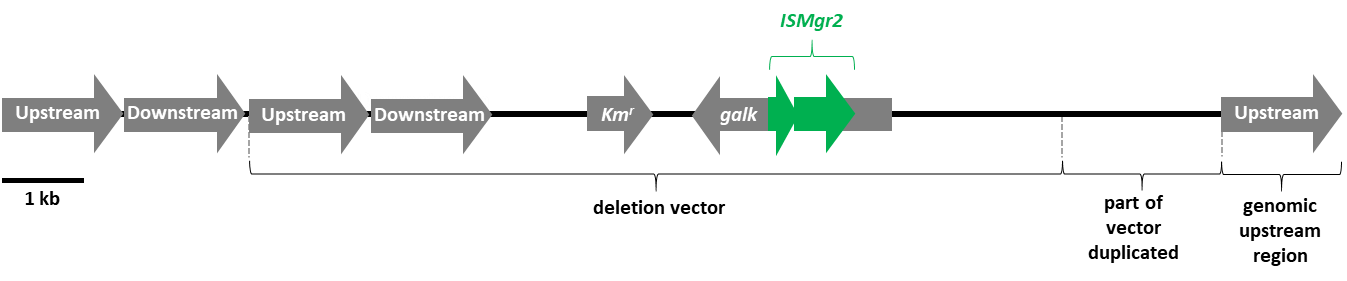


**Figure S3. Genetic organization of the Km^r^, false positive mutant** ∆TZ-15∆M04 K752 Km^r^. The targeted M04 had not been deleted but was still maintained in the genome. A large part (~9.1 kb) of the 10.2 kb deletion vector *pORFM-GalK-M04* harboring the *Km^r^* gene was found to be inserted at the intended site, but harboring a spontaneous duplication of both the upstream and downstream homologous regions intended for targeted insertion of the deletion construct by homologous recombination. In addition, the *galK* gene was inactivated by insertion of a copy of the IS element *ISMgr2* into the central region.


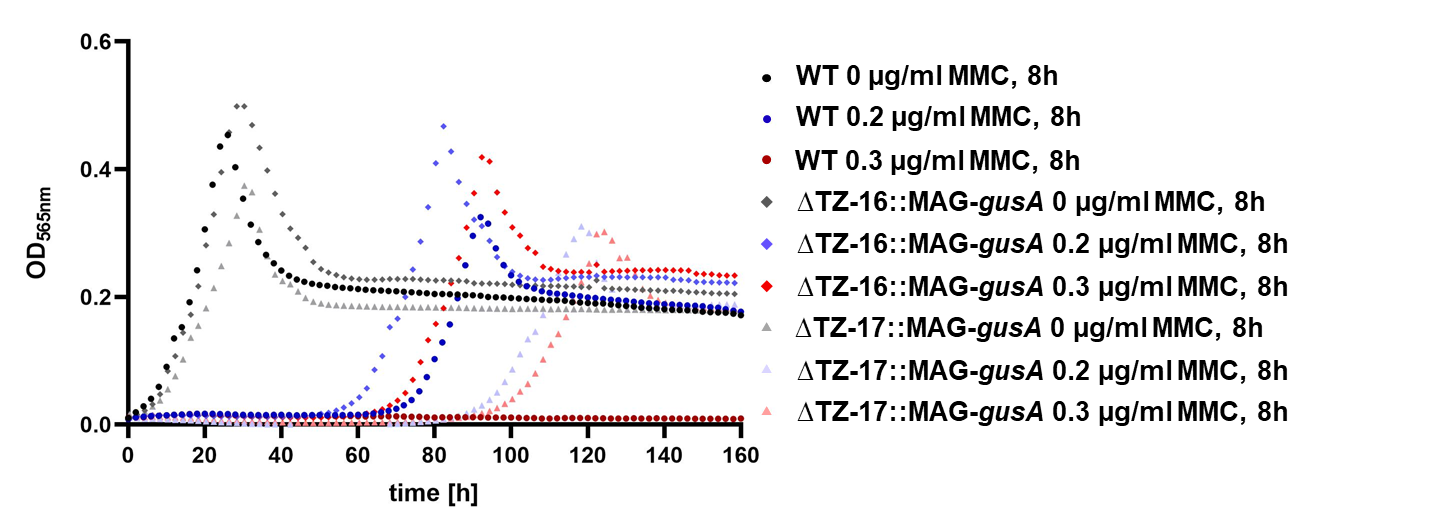


**Figure S4.** Growth profiles of these strains induced with MMC with concentrations between 0.2–0.3 µg/ml MMC, induced 8 h. Cells were washed twice, adjusted to initial OD and growth experiments started at 28°C under aerobic conditions and each strain was analyzed in triplicates while each curve shows its average (standard deviation <5%).


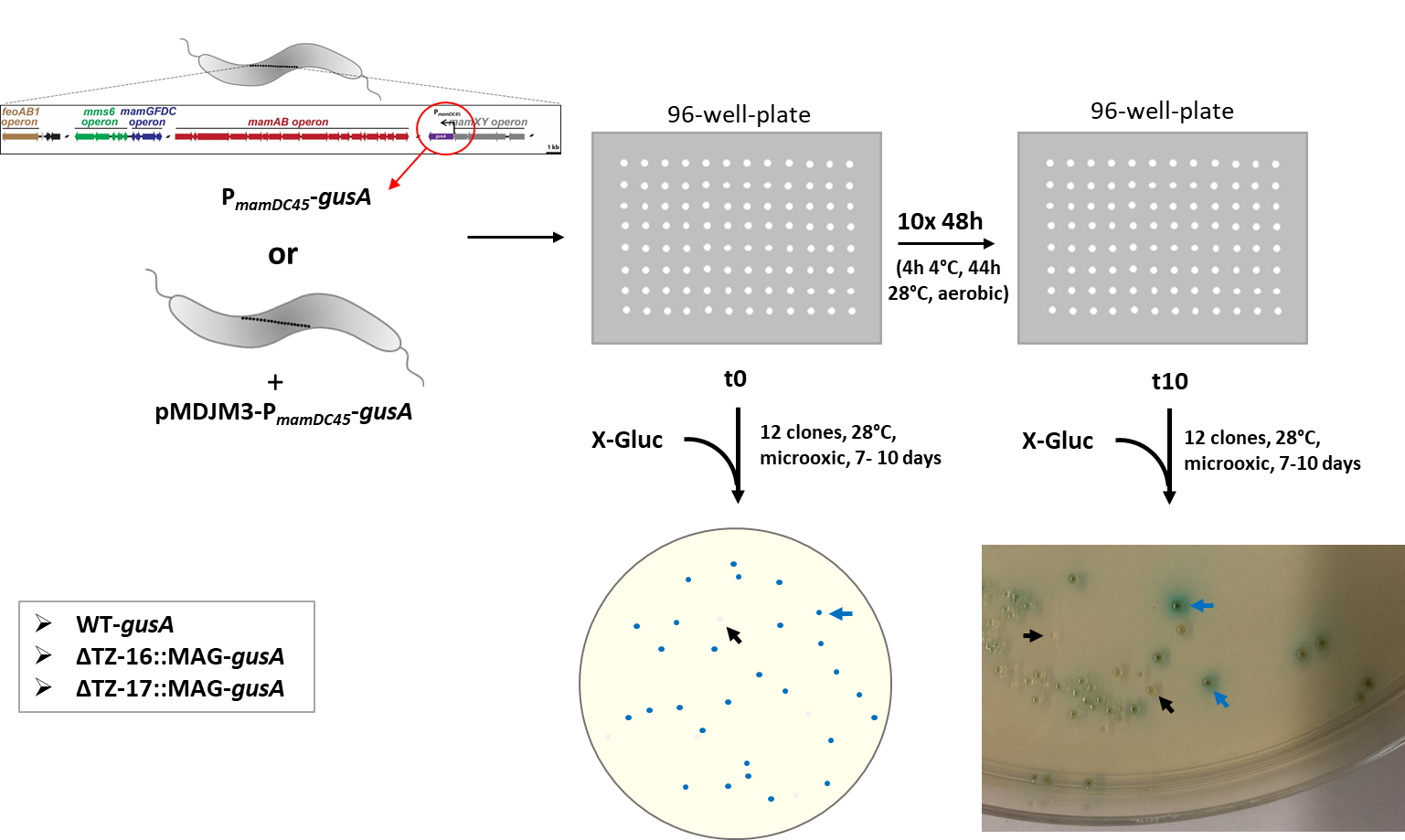

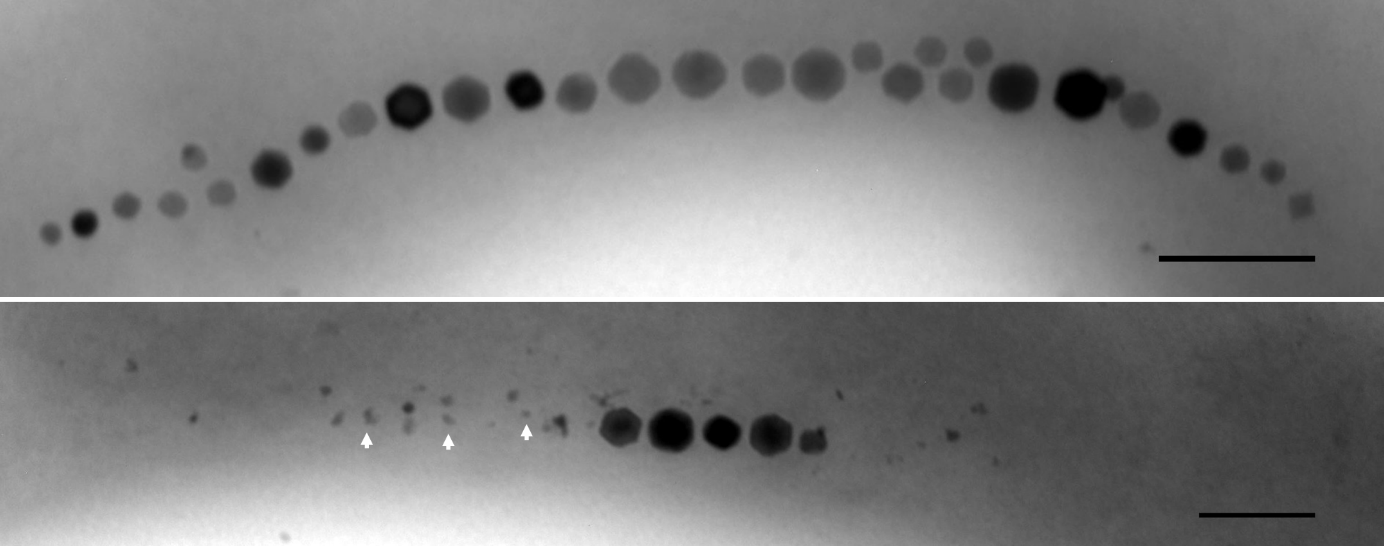


**B**

**A**

**Figure S5. Experimental procedure of the genetic stability assay (A) and identified magnetosome phenotypes (B).** Blue arrows indicate blue colonies while white/brown colonies are marked by black arrows **(A)**. TEM micrographs **(B)** show WT-like magnetosome chains (upper micrograph) and flake-like particles (lower micrograph, white arrows) could be observed in several of WT-*gusA* clones after ten passages. Scale bars: 100 nm.
